# Shape analysis of the basioccipital bone in *Pax7*-deficient mice

**DOI:** 10.1038/s41598-017-18199-9

**Published:** 2017-12-20

**Authors:** Joshua Cates, Lisa Nevell, Suresh I. Prajapati, Laura D. Nelon, Jerry Y. Chang, Matthew E. Randolph, Bernard Wood, Charles Keller, Ross T. Whitaker

**Affiliations:** 1 Scientific Computing and Imaging Institute, University of Utah, Salt Lake City, UT USA; 2 Department of Anthropology, Center for the Advanced Study of Human Paleobiology, The George Washington University, Washington DC, USA; 3 Greehey Children’s Cancer Research Institute, University of Texas Health Science Center, San Antonio, TX USA; 4 Children’s Cancer Therapy Development Institute, Beaverton, OR USA

## Abstract

We compared the cranial base of newborn *Pax7*-deficient and wildtype mice using a computational shape modeling technology called particle-based modeling (PBM). We found systematic differences in the morphology of the basiooccipital bone, including a broadening of the basioccipital bone and an antero-inferior inflection of its posterior edge in the *Pax7*-deficient mice. We show that the *Pax7* cell lineage contributes to the basioccipital bone and that the location of the *Pax7* lineage correlates with the morphology most effected by *Pax7* deficiency. Our results suggest that the *Pax7*-deficient mouse may be a suitable model for investigating the genetic control of the location and orientation of the foramen magnum, and changes in the breadth of the basioccipital.

## Introduction

The role of *Pax7* in patterning fetal craniofacial features was first described by Mansouri and colleagues^1^. Homozygous *Pax7*-deficient mice typically died within 2 weeks of birth. Disrupting both copies of *Pax7* during embryogenesis resulted in underdevelopment (antero-posterior shortening) of the maxilla and lacrimal bones. The craniofacial defects observed in *Pax7*-deficient mice were restricted to neural crest derived portions of the skull, and skeletal preparations showed apparently normal morphology at the cranial base sutures at P0. Mansouri *et al*.^1^ also reported a reduced number of tubules in the serous glands of the nose, and the inferior lateral part of the nasal cavity is missing; both nose phenotypes were attributed to neural crest defects. Mansouri *et al*.^1^ report no gross morphological abnormality in the neuronal derivatives of the cephalic neural crest. The *Pax7*-deficient mouse was later found to have a compelling defect in postnatal muscle growth^2^, which was thereafter carefully clarified as a defect in postnatal muscle stem cell renewal^3^, but not prenatal muscle patterning, therefore making it unlikely that *Pax7* mutant craniofacial defects could be attributed to factors other than cranial patterning. However, the role of *Pax7* in chondrocranium development has been largely unexplored.

Several mouse models alter the expression of genes implicated in cartilage maturation (e.g., *FGFR3*), are known to cause premature fusion of cranial base sutures, antero-posterior shortening of the cranial base, reduction in the size of the foramen magnum, and impaired ossification in the frontal bone^4^. Dexamethasone (Dex) is commonly used in cancer treatment, but has also been shown to alter FGF signaling, repress *Pax7* expression, and lead to disrupted cranial neural crest development^5^. The *FGF* signaling cascade has been shown to be necessary and sufficient to induce *Pax3*, but did not induce *Pax7* expression after neural crest cells complete their migration towards the face^6^. The *Pax7*-deficient mouse provides a model with which to test whether *Pax7* deficiency is sufficient to induce antero-posterior shortening of the cranial base.

The statistical shape analysis approach used in this study is called particle based-modeling (PBM). PBM is a technique for examining the phenotype of a population that uses a point-correspondence model to represent homology across anatomical surfaces. In a point-correspondence model, anatomical geometry is represented by many hundreds or thousands of landmark points called “correspondences”. In the PBM approach correspondence points, which are computed automatically, allow for a very detailed description of the geometric variability of anatomical structures, especially when that geometry is derived from high-resolution three-dimensional (3D) imaging, such as microCT. PBM computes correspondences through an optimization process that seeks to minimize the information content (entropy) of the resulting shape model, thus maximizing its statistical power, relative to arbitrary descriptions of shape. The latter is an important consideration for the investigation of subtle and complex questions of phenotype. PBM has been described in a series of papers in the computer science literature^7–11^ and has been applied to neurobiology, orthopedics, and clinical cardiology^11–17^.

Our study examines the hypothesis that the cranial base of the *Pax7*-deficient mouse may provide a tractable model with which to investigate the genetic control of foramen magnum location and orientation and basioccipital shape. We compared the basioccipital bone in newborn mice heterozygous or homozygous for *Pax7* deficiency with age-matched wildtype mice of a similar strain background (C57BL/6). Our analysis describes significant shape differences among cohorts, which are supported by empirical observations of volume renderings of microscopic computed tomography (microCT) imagery of the anatomy for each group. Although *Pax7*-deficient mice do not show a significant difference in length of the basioccipital bone along the sagittal plane, parasagittal length of the basioccipital is increased in *Pax7*-deficient mice. *Pax7*-deficient mice have broader basioccipital bones, with increased mediolateral breadth around the paired lateral synchondroses. The anterior margin of the foramen magnum is more concave in *Pax7*-deficient mice, resulting in a more anterior placement of basion with respect to the basioccipital bone. *Pax7*-deficient mice also exhibit a more inferior-posterior orientation to the foramen magnum.

## Results

**Figure 1:**
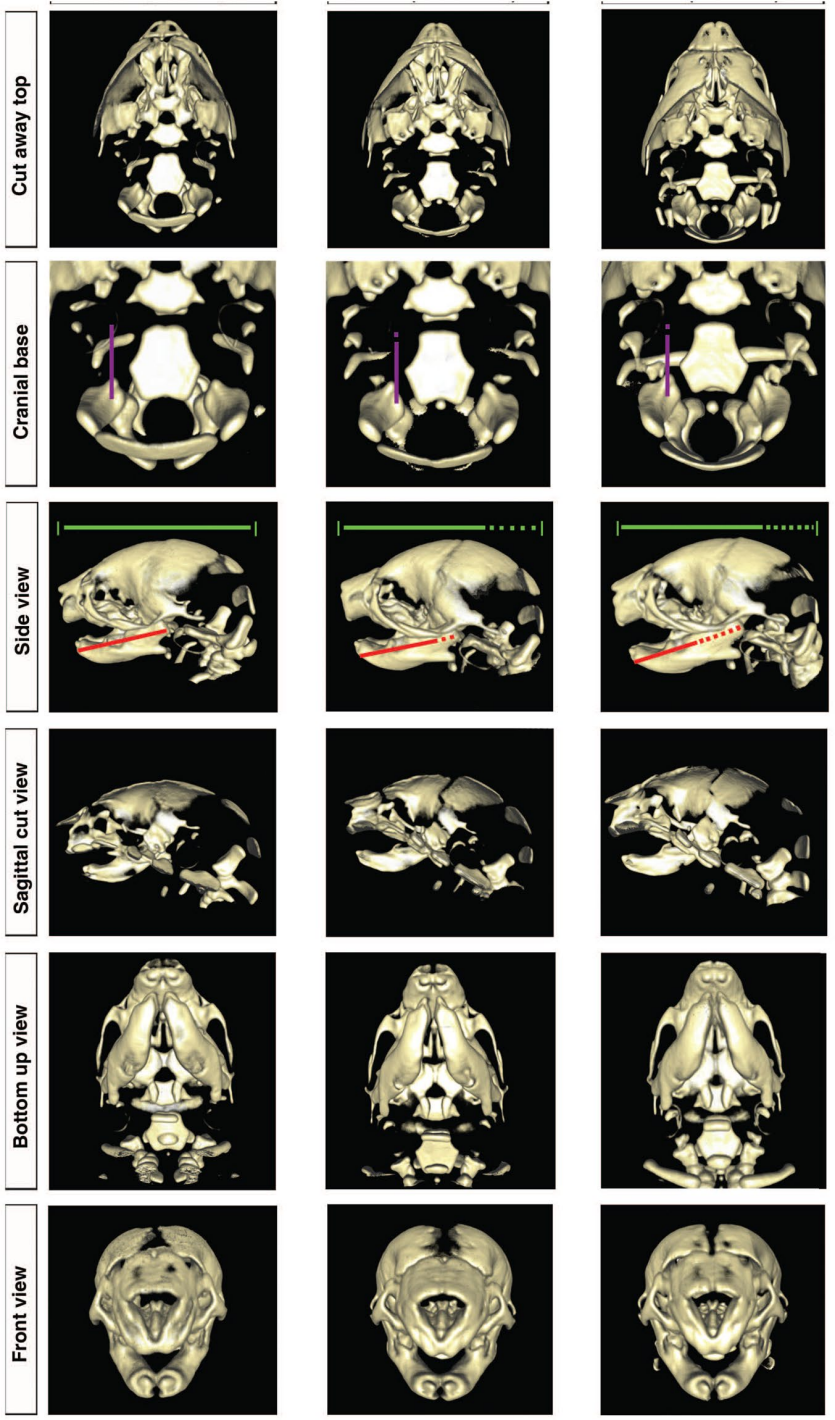
Volume rendering visualizing the microCT scans for specimens with median skull length from the wildtype *Pax7* ^(*WT/WT*)^, homozygous *Pax7* ^(*LacZ/LacZ*)^ and heterozygous *Pax7* ^(*LacZ/WT*)^ groups. Row A depicts the neonatal skull base, with the temporo-parietal bones cropped away. Row B is a closer view of the cranial base and the basioccipital bone. Row C compares the overall jaw and skull lengths. Side-view images for the sagittal hemi-sections of each sample are shown in Row D and images looking onto lower jaws from bottom are given in Row E. Row F compares craniofacial features of the front side of the skull. These images were created using the freely available open source ImageVis3D volume rendering software (ImageVis3D, http://www.sci.utah.edu/cibc/software). The jaw lengths and overall skull lengths were calculated using MicroView http://microview.sourceforge.net/ (GE Healthcare, London, Ontario, Canada) such that three dimensional landmark coordinates were collected from two dimensional images. Each dash in the scale bar reflects a 0.1 mm increase.

The prenatal disruption of *Pax7* function results in subtle craniofacial changes. In this study, we used *Pax7* ^*LacZ*^, whereby *LacZ* replaces the normal (wildtype) *Pax7* gene. Volume-rendering visualizations of the microCT scans for representative specimens from the wildtype *Pax7* ^*WT/WT*^, homozygous *Pax7* ^*LacZ/LacZ*^ and heterozygous *Pax7* ^*LacZ/WT*^ groups are shown in Fig. 1. The figure depicts the neonate animal with the median skull length from each group. Key phenotypic differences in Fig. 1, by inspection, include lengthening of the mandible and an increase in the overall anteroposterior length (Row 3). The absolute changes in each case were more pronounced for heterozygous *Pax7* ^*LacZ/WT*^ mutant animals than homozygous *Pax7* ^*LacZ/LacZ*^ mutant animals.

**Figure 2:**
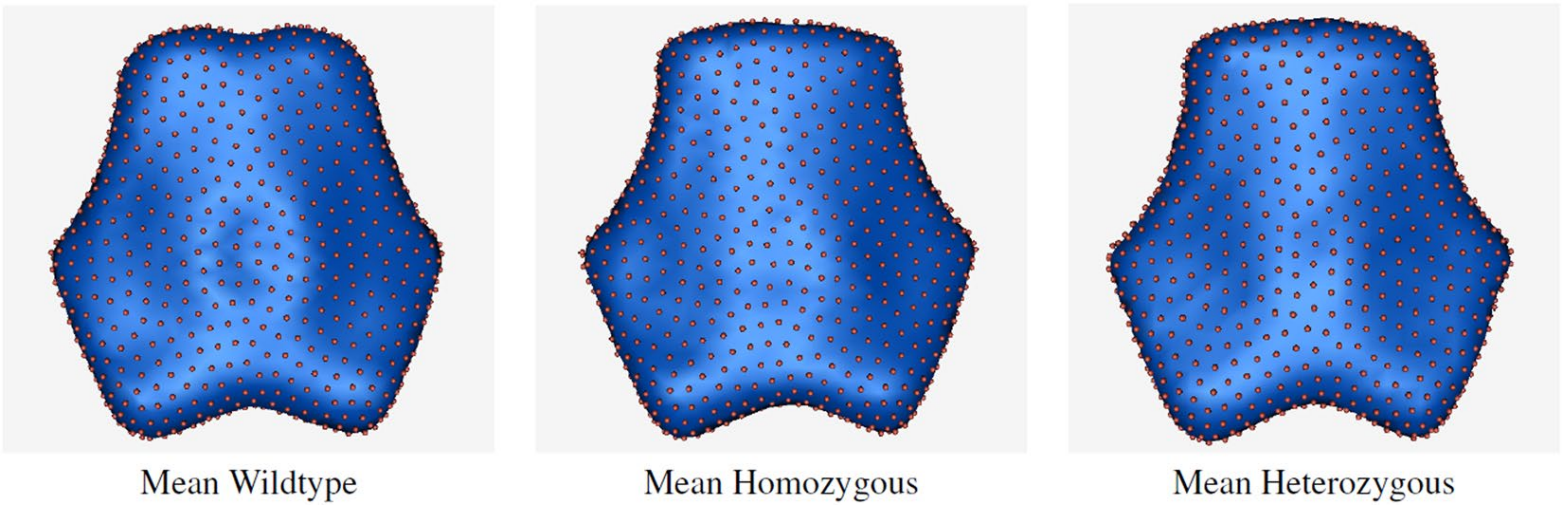
A visual comparison of the group mean shapes. Shape surfaces are reconstructed from the Euclidean means of the correspondence positions for each group. Mean correspondence positions are indicated by spheres. (**A**) wild type, (**B**) homozygous, (**C**) heterozygous.

For subsequent analyses of the cranial base, we concentrate on the third phenotypic feature of *Pax7* deficient animals, a change in shape and anteroposterior (parasagittal) length of the basioccipital bone (Row 2), as measured by PBM analysis. The mean PBM shape for each group in our study cohort is shown in Fig. 2 (superior view). These shapes are surface reconstructions computed from the average surface-point correspondence configurations within each group. Correspondence points are shown as red spheres, and the surface reconstructions were computed using the algorithm described in Hoppe, *et al*.^18^. Note the similarity between the homozygous and heterozygous mean shapes, and the relative contrast with the wildtype shape.

**Figure 3:**
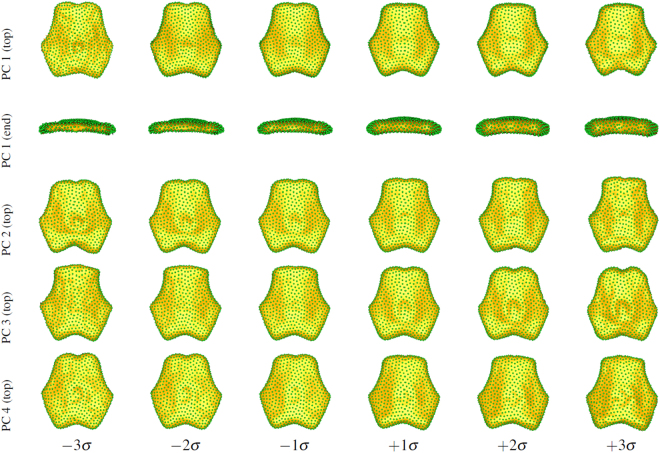
Variation in each of the top 4 principal components of the homozygous basioccipital model. Shape (yellow surface) is reconstructed from correspondence positions (green points) at 1, 2, and 3 standard deviations (*σ*) from the mean shape. (**A**) PC1 top, (**B**) PC1 end, (**C**) PC2, (**D**) PC3, (**E**) PC4, (**1**) −3 *σ*, (**2**) −2 *σ*, (**3**) −1 *σ*, (**4**) 1 *σ*, (**5**) 2 *σ*, (**6**) 3 *σ*.

Figure 3 illustrates the shape variation described by each of the principal component modes in the homozygous model at 1, 2 and 3 standard deviations from the mean. Variation in the heterozygous model modes is very similar and omitted here for brevity. Table 1 summarizes the results of the multivariate Hotelling *T* ^2^ statistical test for differences in the mean shapes from Fig. 2, along with the number of PCA loadings (determined by parallel analysis^19^) for the dimensionality reduction. For the homozygous and heterozygous models, the results indicate highly significant shape differences from the wildtype group (*p* ≪ 0.001) when considering both the parametric *T* ^2^ distribution and a nonparametric distribution estimated with 20,000 random group permutations. No significant differences between homozygous and heterozygous groups are indicated with either test.

**Table 1:** Multivariate hypothesis test results for group differences in mean shape for *Pax7* ^(*LacZ/LacZ*)^, *Pax7* ^(*LacZ/WT*)^, and combined mutant groups.

*Correspondence model*	# PC modes	*Hotelling T* ^*2*^ *parametric test*	*T* ^*2*^ *permutation test*
Homozygous (*LacZ/LacZ vs WT*)	5	*T* ^2^ = 95.33, p ≪ 0.001	p ≪ 0.001
Heterozygous (*LacZ/WT vs WT*)	4	*T* ^2^ = 150.16, p ≪ 0.001	p ≪ 0.001
Combined mutant (*LacZ/LacZ vs LacZ/WT*)	4	*T* ^2^ = 1.54, p = 0.852	p = 0.695

**Figure 4:**
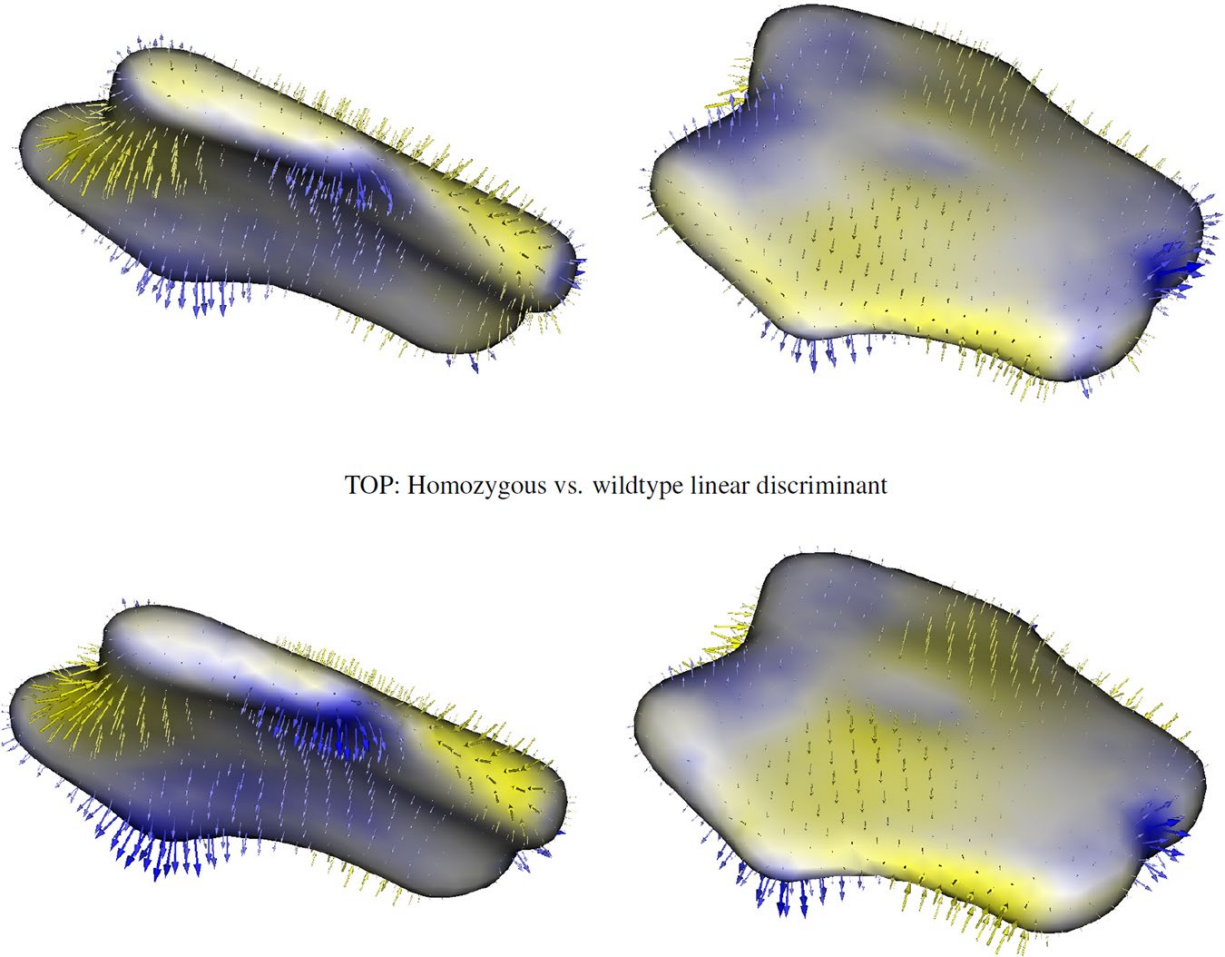
Visualization of the linear discriminant from the Hotelling *T* ^2^ test for the homozygous shape model (TOP ROW) and the heterozygous shape model (BOTTOM ROW). Arrows indicate direction from the wildtype distribution to the mutant distribution.

A closer examination of the statistically-significant shape differences between groups, as determined by the Hotelling *T* ^2^ test on the PCA loadings, is given in Fig. 4. This figure shows the difference in mean correspondence positions along Fisher’s linear discriminant line, which is the direction in the PCA shape space of maximal statistical separation between groups and the line along which the *T* ^2^ statistic is computed. Larger arrows indicate local regions that exhibit more shape change between groups and smaller arrows indicate relatively smaller shape differences (scale of the arrows has been exaggerated for visualization purposes). As indicated in the figure, statistically-significant differences in basioccipital shape between wildtype and *Pax7*-deficient mice include antero-inferior inflection of the posterior edge (which forms the anterior boundary of the foramen magnum), increased concavity of the posterior edge, lateral-superior outgrowth of the lateral prominences, anterior elongation in the midline, and flattening of the posterior inferior surface. Note that the group differences between the homozygous and heterozygous cases are similar to those between the homozygous and wildtype cases, though only the latter differences are statistically significant.

The PBM method preserves information about variation within samples. We applied a randomized multivariate Levene test (Table 2) to address whether two samples differ in terms of the amount of spread in multivariate PCA space. Three models were tested using the mean and the median; it has been argued that the median is more robust in univariate analyses and so has been extended to this multivariate case^20^. The homozygous *Pax7* vs. wildtype model (*p* = 0.8), heterozygous *Pax7* vs. wildtype model (*p* = 0.3), and homozygous *Pax7* vs. heterozygous *Pax7* (*p* = 0.5) do not indicate significant differences in variance. The number of PCA modes used in the analysis were selected by parallel analysis.

**Table 2:** Levene tests indicate no significant differences in variance between the *Pax7* deficient and *Pax7* wildtype basioccipital models. Values shown are *p* values using several variations on the Levene test statistic (mean, median, randomized mean, randomized median).

Sample 1 (*n*)	Sample 2 (*n*)	PCA modes	mean	median	randomized mean	randomized median
*LacZ/LacZ* (6)	*WT/WT* (12)	5	0.537	0.802	0.528	0.825
*LacZ/WT* (18)	*WT/WT* (12)	4	0.239	0.338	0.248	0.318
*LacZ/LacZ* (6)	*LacZ/WT* (18)	4	0.432	0.509	0.426	0.502

**Figure 5:**
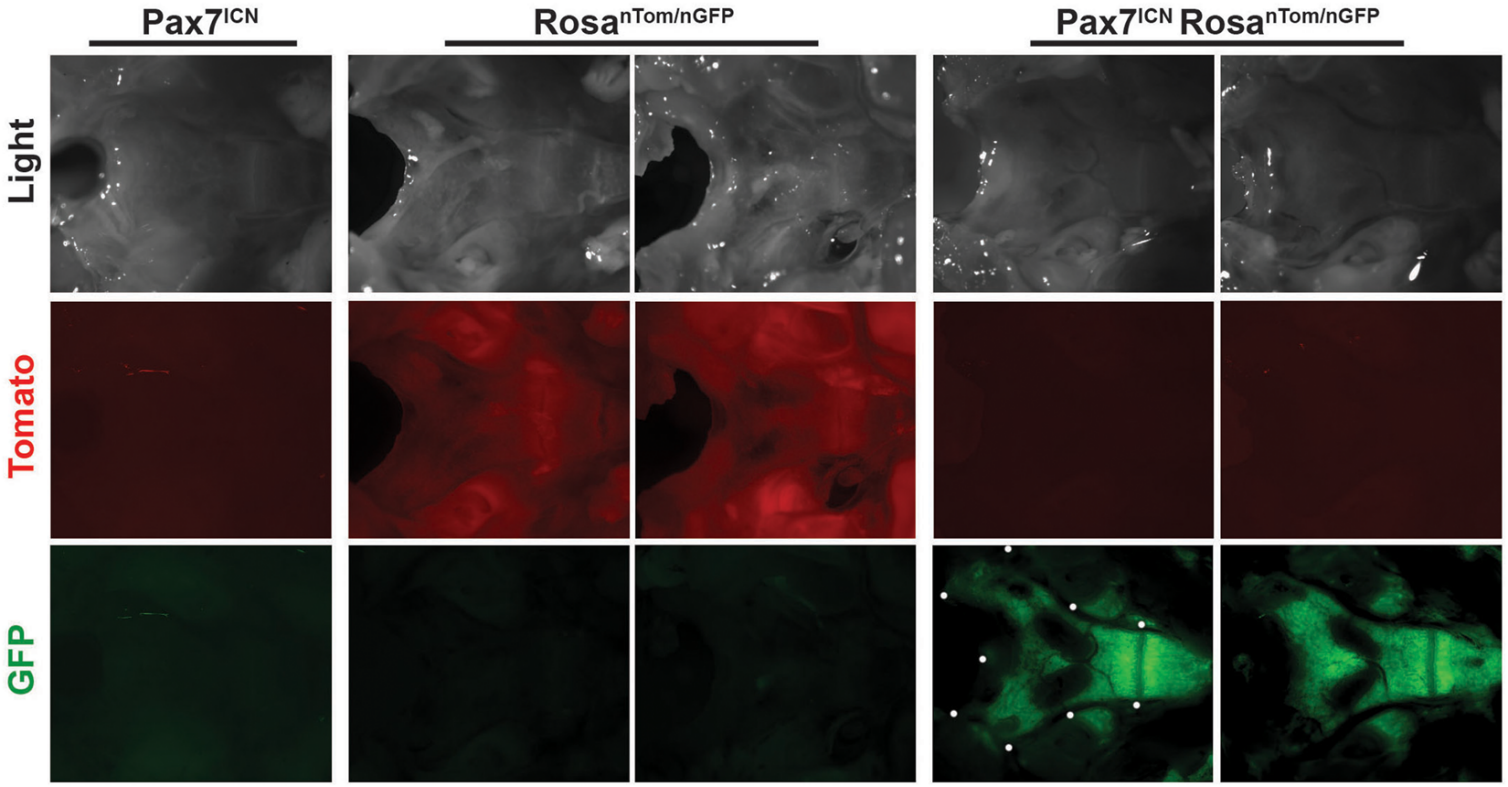
Pax7 expression in the murine basioccipital bone. Representative bright field (1) and fluorescent images (tdTomato, red (2); GFP, green (3)) of the dorsal aspect of basal occipital bones were obtained from 4 to 5-week-old (**A**) *Pax7* ^*ICNm/WT*^ *Rosa* ^*WT/*WT^ (Pax7^ICN^), (**B**) *Pax7* ^*WT/WT*^ *Rosa* ^*nT-nG/WT*^ (Rosa^nTom/nGFP^), and (**C**) *Pax7* ^*ICNm/WT*^ *Rosa* ^*nT-nG/WT*^ (Pax7^ICN^ Rosa^nTom/nGFP^) mice. Rostral region is visualized on the right and the caudal region on the left of each image. Eight mice were analyzed per genotype. White dots denote the anatomical boundary of the basioccipital bone. Eight mice were analyzed per genotype. Three of the eight *Pax7* ^*ICNm/WT*^ *Rosa* ^*nT-nG/WT*^ mice did not demonstrate RFP or GFP fluorescence, which was attributed to transgene silencing for the reporter allele. Images were acquired at 7X magnification.

To understand the role played by *Pax7* gene expression in the determination of basioccipital shape, we conducted *Pax7* lineage tracing using a *Pax7* ^*Cre*^ mouse strain^21^ crossed with a *Cre-LoxP GFP* reporter mouse strain^22^. The design of this mouse model included a small protein made the same time as *Pax7* called *Cre*. Every time *Pax7* was expressed so was *Cre*. *Cre* acted like a pair of scissors that irreversibly excise DNA found between two LoxP cites. The mice also expressed a *Cre* conditional fluorescent protein from all cells. All of the cells that expressed *Cre*, switched from red protein to green protein. The switch was a permanent deletion of the genome and irreversible. Thus, we measured all of the cells and their progeny that ever expressed *Pax7*. Mice were harvested at age 4–5 weeks, and *GFP* expression was measured. *Pax7* was indeed expressed in the basioccipital bone cell lineage (Fig. 5). We compared the black, non-*Pax7* (non-*GFP*) expressing central regions in Fig. 5 to the regions in Fig. 4 showing areas best distinguishing groups (arrow size). The central areas where *Pax7* was not expressed also did not distinguish between groups. Areas with strong GFP expression (Fig. 5) corresponded to areas that distinguish between groups (Fig. 4) at the anterior and lateral synchondroses. The white dots in Fig. 5 indicate the edge of the foramen magnum in the bright field image. The anterior edge of the foramen magnum did not express GFP but was directly adjacent to areas that had strong GFP expression.

## Discussion

Particle-based modeling (PBM) has potential advantages for measuring subtle shape variation over existing shape analysis methods, which include manual landmarking, image warping comparisons, and over methods that establish correspondence through explicit shape parameterizations. Landmark- based methods^23^ require user-identification of shape inflections (corners and edge prominences), which may be similar between wildtype and mutant tissue structures when phenotypes are mild. However, these landmarks assume a priori knowledge of the location of anatomical differences and can be challenging to identify objective landmarks when substantial differences among phenotypes exist. Methods that involve warping one shape onto another (via coordinate transformations)^24^ typically require analysis relative to an atlas or template, and data-driven image atlases^25^ are not yet able to adapt the correspondence to statistical models of the underlying population. Other approaches related to PBM include methods to establish automatic surface point correspondences, including the spherical harmonics point distribution model (SPHARM-PDM)^26^ and minimum description length (MDL) algorithm^27^. Both approaches seek automated surface point correspondences. However, unlike PBM, these methods rely on explicit parameterizations of shape, either as a way to establish correspondence, as with SPHARM-PDM, or to constrain the model optimization^28^. SPHARM-PDM also does not consider the statistics of the sample cohort. The use of SPHARM-PDM to initialize a correspondence model, followed by statistical optimization with PBM, has therefore been proposed to produce more compact and statistically powerful models over SPHARM-PDM alone^29^.

In this study, we applied PBM to the statistical analysis of shape changes to the mouse cranial base in the absence of *Pax7*. While we observed qualitative differences in the mandible and anteroposterior dimension, this analysis focused on statistically-significant differences in basioccipital shape that influence the placement of the foramen magnum. In our study, mice deficient in *Pax7* showed an antero-inferior deflection of the rostral aspect of the foramen magnum, as well as lateral-superior outgrowth of the lateral prominences.

A number of questions remain to be answered by future studies of the role of *Pax7* in the patterning of the basioccipital bone in mammals before birth. One question is whether *Pax7* is expressed in the chondocranium of the fetus, thereby influencing the basioccipital shape directly, or whether craniofacial or brain growth are more indirect influences on basioccipital shape. Our lineage tracing of the basioccipital answers this question to some degree by affirming that the basioccipital itself expresses *Pax7* and that the areas of greatest shape difference in *Pax7* haploinsufficient or nullizygous mice are within or directly adjacent to tissues belonging to the *Pax7* lineage. Nonetheless, prior studies support the hypothesis that facial hafting and brain size are highly integrated with cranial base growth and development^30^. Both our analysis and the overview of the *Pax7* phenotype presented by Mansouri and colleagues^1^ are in agreement that maxillofacial changes exist. In addition to responses to brain size and facial hafting, the cranial base responds to growth along synchondrosis^4^. We examined the patency of basicranial synchondroses in later stages of development to address the question of whether *Pax7* directly impacts basioccipital morphology through premature fusion of synchondroses. The exoccipital-basioccipital (EO-BOS) synchondroses and sphenoccipital synchondroses (SOS) are not fused in the *Pax7* deficient phenotype at the time of our genetic proof of concept study at P0, P6, P27, but as expected are fused at 6 months (data not shown).

For future studies, *Pax7* expressing lineage tracing of the E13.5-E15 chondocranium remains to be evaluated, and fortunately new tools enabling the dissection of cranial neural crest and cranial mesenchyme at the level of specific enhancer elements are now available^31^. Future studies should also consider the possibility that shape modifications in the *Pax7* deficient basioccipital are a secondary phenomenon through analysis of cre mediated *Pax7* knockout models. Previous work on the modularity of skull morphogenesis shows that chondrocranial development and brain development interact. The midbrain morphology of the *Pax7* deficient mouse remains undescribed, however the entirety of the midbrain is within the *Pax7* expressing lineage^32^. Future studies should assess midbrain size and shape in the *Pax7* deficient mouse model and what (if any) influence midbrain morphology has on basioccipital shape. One salient paper has demonstrated that interactions between brain size, face size, and the widths and lengths of the components of the cranial base account for a large percentage of variation in cranial base angle in mice^30^. The *Pax7* deficient mice may provide a tractable animal model to investigate the genetic control of basioccipital proportions, foramen magnum location and orientation, and morphological integration between the basioccipital bone, neural crest derived portions of the face, and the midbrain.

### The Role of Pax7 in Basioccipital Development and Human Evolutionary Developmental Biology

One of the features that distinguishes hominins (i.e., modern humans and their close relatives) from the extant apes is the adoption of an upright posture and a bipedal gait and enlargement of the brain. In the cranium, one consequence of these changes is the more central location of the foramen magnum^33,34^. One of the ways a central foramen magnum is achieved is by a shortening of the basioccipital part of the basicranium^35^. The length, breadth, location, and orientation of the foramen magnum (influenced by basioccipital morphology) have been part of recent debates about the hominin status of *Sahelanthropus tchadensis* ^36^ and *Ardipithecus ramidus* ^37,38^. Evidence from the posterior basicranium has also featured in debates about the taxonomic distinctiveness of early *Homo* ^39^, the relationship between *Paranthropus boisei* and *Paranthropus aethiopicus* ^40^, and in discussions about the nature of the relationships between *Paranthropus* and early *Homo* ^41^. The conserved developmental genetic pathways which play a role in cranial base development, particularly in the basioccipital bone, must be understood in order to effectively interpret morphology observed in the hominin fossil record and in particular to evaluate hypotheses that suggest such changes are homoplasic.

This paper does not claim that changes in *Pax7* expression or function affecting the basioccipital resulted in the basicranial modifications we see in the hominin clade. Instead we argue the broader point that understanding craniofacial development can inform our interpretation of the taxonomic significance of morphology observed in the hominin fossil record. The morphology of the foramen magnum depends on many interconnected factors, the shape of the basioccipital being one of these factors. Furthermore, the shape of the basioccipital bone itself is under the influence of many local and regional factors. While this study focused on the shape of an isolated bone, future studies will look more closely at how the basioccipital bone phenotype covaries with craniofacial growth in other parts of the *Pax7* deficient skull. For example, synchondroses are one of the major influences on skull base growth, and we have identified these as locations that are part of the *Pax7* cell lineage and *Pax7* deficient phenotype. Future studies should assess whether cartilage maturation is appropriately organized or timed in the absence of *Pax7* expression.

Evolution is generally parsimonious, but we now know of many examples of complex structures evolving homoplasically. Parsimony suggests that it is unlikely that complex correlated changes in the basicranium would have occurred more than once in the hominin lineage. Paleoathropologists have generally been reluctant to entertain the possibility that an upright posture, a bipedal gait and larger brains might have evolved more than once in the hominin lineage. When paleoanthropologists find a novel taxon with a short broad cranial base they automatically attribute the taxon to the hominin lineage and not the panin lineage. However, the complexity of a structure does not guarantee that this structure has evolved only once in the phylogenetic history of a group. For instance, numerous fossils tend to indicate that the middle ear of mammals (a highly ‘complex’ structure) may have evolved at least three times independently from the proximal end of Meckel’s cartilage. Moreover, the *Pax7* deficient mouse model achieves a hominin-like constellation of coordinated morphological innovations with a change to the expression level of a single gene. In fact, *Pax7* deficiency is only one of several mouse models that alter the anterior-posterior length of the basicranium^4^. These findings support a view of hominin evolution that predicts that basioccipital morphology is evolvable, predicts some degree of homoplasy in the basioccipital, and cautions against allowing a short-broad basioccipital or anteriorly placed foramen magnum alone to attribute taxa to the hominin lineage.

The changes to the basioccipital in the *Pax7*-deficient mouse are analogous to those that occur in human evolution. The cranial base of the *Pax7*-deficient mouse differs from that of the wildtype mouse, in ways that recall the differences between the cranial base of modern humans differs from that of our closest living relatives, common chimpanzees and bonobos. These changes include antero-inferior deflection of the rostral portion of the foramen magnum, growth at the lateral prominences and relative broadening of the basioccipital. *Pax7* deficient mouse phenotypes resemble features of the cranial base (e.g., the anterior placement and inferior orientation of the foramen magnum, and greater breadth of the basioccipital bone) that distinguish hominins from panins. Furthermore, the pleiotropic effects of *Pax7* deficiency coordinate maxillofacial reductions with changes in basioccipital morphology. These results should focus attention on whether modern humans, and chimpanzees/bonobos regulate *Pax7* gene expression differently.

## Experimental Procedures

### *Pax7* Knockout Mice

Mice with a *LacZ* gene insertion interrupting the native *Pax7* gene have been previously described^3^. The homozygous mutant sample (n = 6) and the heterozygous mutant sample (n = 18) were each drawn from the same litters. These mice were maintained on a mixed strain background that was greater than 75% C57BL6. Wildtype C57BL6 mice (n = 12) were taken from separate litters. Wildtype C57BL6 mice were purchased from Taconic Farms (Hudson, NY). Mice at postnatal day 0 pups were euthanized by isoflurane inhalation. Carcasses were preserved in 10% buffered formalin. All animal procedures were conducted in accordance with the Guidelines for the Care and Use of Laboratory Animals and were approved by the Institutional Animal Care and Use Committee at the University of Texas Health Science Center at San Antonio and cc-TDI.

We performed genotyping from tail snips of euthanized mice. Tail tip DNA was prepared as previously described. For PCR-based genotyping, the following primers were used to test for the *Pax7* deletion: (ck291) 5-GTCGGGTCTTCATCAACGGTC-3, (ck292) 5-GGGCTTGCTGCCTCCGATAGC-3, and (ck293) 5-CGCGCTCGAGATGTGCTGCAAGGCGATTAA-3. Our thermocycling protocol was 95 °C for 5 min, 95 °C for 30 sec, 58 °C for 30 sec, 72 °C for 50 sec, repeat cycle (n = 30), 72 °C for 7 min, 10 °C thereafter. Samples were analyzed on a 1X TAE 2.5% agarose gel by electrophoresis and visualization with ethidium bromide. The *Pax7* ^*LacZ*^ allele band size is (240 bp), the wildtype band size is (200 bp).

### Lineage Tracing

To assess *Pax7* expression in the basal occipital bone, *Gt(ROSA)26* ^Sortm1(CAG-tdTomato*,-EGFP*)Ees^ (*Rosa* ^*nT-nG*^, The Jackson Laboratory, Bar Harbor, ME) homozygous females were bred with homozygous *Pax7* ^*tm1(cre)Mrc*^ (*Pax7* ^*ICNm*^) males to generate F1 *Pax7* ^*ICNm/WT*^, *Rosa* ^*nT-nG/WT*^ mice. F1 mice were crossed to generate the experimental F2 cohorts of *Pax7* ^*ICNm/WT*^, *Rosa* ^*WT/WT*^, *Pax7* ^*WT/WT*^, *Rosa* ^*nT-nG/WT*^, and *Pax7* ^*ICNm/WT*^, *Rosa* ^*nT-nG/WT*^. The nature of this reporter is that Cre expression converts baseline red reporter gene fluorescence to green reporter gene fluorescence. Four- to five-week-old male and female F2 experimental mice were euthanized via CO_2_ asphyxiation. Following post-mortem decapitation, the skin, cranium, brain tissue, and ventral muscle tissue were removed to expose the basal occipital bone for imaging. Bright field and fluorescent images were acquired at 7x magnification using an AXIO Zoom.V16 microscope (Carl Zeiss MicroImaging, Oberkochen, Germany) with a PlanNeoFluar Z 1x/0.25 FWD 56 mm objective lens and Axiocam 503 mono camera (Carl Zeiss MicroImaging). ZEN 2.3 (blue edition) software (Carl Zeiss Microscopy GmbH, Jena, Germany) was used to electronically acquire experimental images. All images were globally processed for size and brightness using Adobe Photoshop CC 2015.0.1 (Adobe, San Jose, CA).

### MicroCT Segmentations

The neonatal mice specimens for our *Pax7* study were imaged by microscopic computed tomography (microCT) at 27 *µ*m isometric resolution using a GE eXplore Locus RS-9 volumetric microCT scanner (GE Healthcare, London, Ontario, Canada). The mouse pups were scanned with the following parameters: 720 views, 10 frames per view, 55 kVp energy, 500 *µ*A current, and 2500 ms exposure time. We performed volumetric segmentations of each basioccipital bone from the CT image volume using a standardized process. The segmentation process proceeded as follows. First, the observer selected an intensity threshold that (subjectively) best separated bone in the CT image from the background. Because of the quantitative nature of CT and the high contrast between bone and tissue, this process is fairly robust to the specific choice of a threshold value. Next, the specific boundaries of the basioccipital bone, relative to surrounding bone structures, were manually delineated by two independent expert observers (L.N. and J.Ch.). The software we used for the segmentation process is the freely available open source Seg3D image processing tool (Seg3D^31^, http://www.sci.utah.edu/cibc/software).

### Particle-Based Modeling (PBM) of Shape

To quantitatively describe the shape of the basioccipital bone, we used a specific type of shape model called a point-based model. Point-based models are a computational extension of a family of widely-used morphometric analysis techniques that involve identification of corresponding landmark positions on a collection of shape samples^23,42^. In contrast to manually- determined landmark models, computationally-derived point-based models consist of dense sets of hundreds or thousands of landmarks that are computed automatically. These dense sets of landmarks are called “correspondence points” and can model shape geometry in much higher detail than traditional manual approaches. Correspondence points can be used for statistical analysis in a variety of standard multivariate approaches^43,44^.

To compute the correspondence points for our shape models of the basioccipital bone, we use a specific optimization algorithm called “particle-based modeling” (PBM). The development of PBM is described in a series of papers from our laboratory^11–15^ and has proven effective for investigation of scientific and clinical questions in a range of applications including neuroscience^11,13,14,17^, biological phenotyping^16,45^, and orthopedics^15,16^. PBM represents the correspondence points as interacting sets of particles that redistribute themselves under an energy optimization. The optimization finds correspondence positions that minimize the entropy of the model, which is a metric of information content. By minimizing information content, PBM learns the shape parameters that are the *most efficient descriptors* of the geometry of the LA, thereby maximizing the model’s statistical power and generalizability. For our study, we used an open source distribution of the PBM algorithm called ShapeWorks (http://www.sci.utah.edu/software.html), which was developed at the University of Utah.

### PBM Shape Parameters and Statistical Analysis

The mathematics and theory behind point-based shape models have been developed over the last several decades and are described in many excellent reference texts and papers, for example^44,46–48^. Here we briefly summarize the major concepts that are relevant to the results presented in this paper. We define a point-based shape model as a collection of *n* sets of *k* correspondence points (3D landmark positions). In our case, *n* = 36 represents the number of basioccipital bone segmentations and *k* = 1024 is the number of correspondence points placed on each one. Thus, each basiooccipital surface geometry is represented by a unique set of *k* *=* 1024 3D points. Correspondence among the segmentations is determined by running the PBM algorithm to produce a set of *k* correspondence points *x*. Point *x*_*i*_ on segmentation number 1 corresponds to point *x*_*i*_ on segmentations 2, 3, 4, *…*, *n*, where *i* *=* 1, *…*, *k*. Note that increasing *k* allows for a more detailed representation of shape, while decreasing *k* would produce a model that is more coarse. The value *k* *=* 1024 was chosen empirically as more than sufficient to represent geometric detail of the basioccipital bone.

The average shape geometry in a point-based model is defined as the set of averages of each of the *k* correspondence points. Similarly, the variability in geometry can be described by the variability in each of the *k* correspondence points. All shapes in the model are normalized with respect to scale, such that the root mean square distances of the correspondence points to their centroids are equal to one^44^. Note that normalizing with respect to scale means we are analyzing the geometric variability that remains *after scale is removed*. Our intention is to perform an analysis that is independent from uniform volume change. We can thus interrogate whether Pax7 deficient mice have a different sized basioccipital bone as an independent question from whether they have a different shaped basioccipital bone.

The geometric variability of a PBM point model can be summarized as a set of shape parameters that are the orthogonal directions of a principal components analysis (PCA) of the correspondence point positions. A complete mathematical description of this process can be found in, for example^44,49^. PCA-based shape parameters allow us to compress the very large amount of geometric information into a much smaller representation of shape that is suitable for traditional statistics, while still retaining most of the geometric information of the shapes. Typically, we choose a finite number of shape parameters *m* for analysis either empirically, or by picking a set that accounts for most of the variability in the model. In this work, we use a method called parallel analysis to automatically determine a finite set of PCA modes that are distinguishable from Gaussian noise in the model^19^.

Once the *m* PCA shape parameters are chosen, every basiooccipital bone shape in the cohort can be represented for statistical analysis as an *m*-dimensional vector of scalar values, where *m* is typically less than 10. We also conducted empirical analysis of the variability in shape by reconstructing shapes from arbitrary combinations of different values of *m* parameters. For example, we examined the change in shape described by each PCA parameter when moving between 3 standard deviations from the mean in that parameter (see Fig. 3).

### Visualizing Statistically-Significant Group Differences

Visualization of the results of a statistical group comparison can be important for developmental biologists to affirm the role of a given gene on anatomical patterning. In the case of our PBM model hypothesis tests with the Hotelling *T* ^2^ metric, we propose a direct visualization of the linear discriminant implicit in the test metric.

The Hotelling *T* ^2^, two-sample metric is given by Eq. 1.

1 $$\begin{array}{rcl}{T}^{2} & = & \frac{({n}_{a}{n}_{b})({n}_{a}+{n}_{b}-2)}{{n}_{a}{n}_{b}}{({\mu }_{{\boldsymbol{a}}}-{\mu }_{{\boldsymbol{b}}})}^{T}w{\boldsymbol{,}}\\ {\bf{w}} & = & {({{\rm{\Sigma }}}_{a}+{{\rm{\Sigma }}}_{b})}^{-1}({\mu }_{{\boldsymbol{a}}}-{\mu }_{{\boldsymbol{b}}}),\end{array}$$

where *µ*_**a**_ and *µ*_**b**_ are the means, Σ_*a*_ and Σ_*b*_ are the covariances, and *n*_*a*_ and *n*_*b*_ are the sample sizes of the two groups, respectively. The discriminant vector **w** (also known as Fisher’s linear discriminant) is the line along which the between-group variance is maximized with respect to within-group variance^50^. The Hotelling *T* ^2^ metric is therefore a scaled projection of the group difference onto the discriminant line. Significant group differences are therefore revealed by transforming **w** back from PCA space into the full-dimensional shape space, *i*.*e*. $\hat{{\bf{w}}}={{\bf{E}}}^{-1}\,\tilde{{\bf{w}}}$, where $\tilde{{\bf{w}}}$ is **w** padded to *n*-dimensions with *n k* zeros. The components of the *dM*-vector $\hat{{\bf{w}}}$ can then be mapped onto a visualization of mean correspondence point positions. For this study, we reconstruct a surface mesh from the correspondence points of group *a* and overlay glyphs illustrating the components of the discriminant vector.
